# Gender based differences in drug eluting stent implantation - data from the German ALKK registry suggest underuse of DES in elderly women

**DOI:** 10.1186/s12872-017-0500-y

**Published:** 2017-02-27

**Authors:** Martin A. Russ, Christian Wackerl, Uwe Zeymer, Matthias Hochadel, Sebastian Kerber, Ralf Zahn, Bernhard Zrenner, Hubert Topp, Volker Schächinger, Michael A. Weber, for the ALKK-Study Group

**Affiliations:** 1 Internistische Praxis am Maxplatz, Maxplatz 12, 83278 Traunstein, Germany; 2 Amper-Klinikum, Dachau, Krankenhausstrasse 15, 85221 Dachau, Germany; 3 Institut für Herzinfarktforschung, Bremserstr, 79, 67063 Ludwigshafen, Germany; 4 Medizinische Klinik B - Abteilung für Kardiologie, Bremserstr. 79, 67063 Ludwigshafen, Germany; 5 Herz- und Gefäß-Klinik GmbH Bad Neustadt, Salzburger Leite1, 97616 Bad Neustadt a.d. Saale, Germany; 6 Krankenhaus Landshut-Achdorf, Medizinische Klinik I, Achdorfer Weg 3, 84036 Landshut, Germany; 7 Sana-Klinikum Hameln-Pyrmont, Saint-Maur-Platz 1, 31785 Hameln, Germany; 8 Klinikum Fulda, Medizinische Klinik I, Pacelliallee 4, 36043 Fulda, Germany

**Keywords:** Drug eluting stents, Gender differences, PCI– registry

## Abstract

**Background:**

Observational studies suggest there are gender based differences in the treatment of coronary artery disease, with women receiving evidence based therapy less frequently than suggested by current guidelines. The aim of our study was to evaluate gender based differences in the use of DES.

**Methods:**

We analysed prospectively collected data from 100704 stent implantations in the PCI registry of the ALKK between 2005 and 2009.

**Results:**

The usage of DES increased from 16.0 to 43.9%. Although women had smaller vessel sizes, they received DES less often compared to men (28.2 vs. 31.3%), with an adjusted odds ratio of 0.93 (95% confidence interval 0.89-0.97) at the age of 75, and an adjusted odds ratio of 0.89 (95% confidence interval 0.84-0.94) at the age of 80.

**Conclusion:**

Despite having smaller vessels than men, women were treated less often with DES. These findings apply to women above the age of 75 years. These findings support previous reports, that elderly women with coronary artery disease are treated differently to men.

## Background

Cardiovascular disease remains the leading cause of death in Europe and North-America [1]. Evidence-based treatment of cardiovascular disease, according to European and American guidelines, should not differ between women and men, wether for stable coronary artery disease, acute coronary syndromes or for revascularisation procedures. Nevertheless, gender differences in the treatment of cardiovascular disease is well recognized, with women receiving less evidence-based care. In acute coronary syndromes, women still receive beta-blockers, ace-inhibitors and statins less frequently than men [2]. Furthermore, invasive diagnosis and treatment (by heart catheterisation and PCI), the most effective treatment especially in high risk NSTEMI and STEMI, is withheld from women frequently [3]. One postulated reason for this is the higher age of women at the time of diagnosis and treatment. Another reason is that women are less frequently investigated for coronary disease since the condition is still regarded as a “male” disease. Furthermore, the presenting symptoms of women with coronary artery disease are frequently overlooked, due to their different and so-called “atypical” presentation [4].

Due to their on average smaller height and size, women do have smaller coronary arteries, which may be one reason for inferior results following revascularisation procedures, either Percutaneous Coronary Intervention PCI [5] or Coronary Artery Bypass Grafting [6]. Given the substantially higher risk of restenosis in smaller vessels [7, 8] the attraction of DES in reducing target lesion revascularisation [9–13], should mean a higher usage of DES in women is warranted.

To evaluate possible gender differences, we analysed the ALKK-PCI registry for gender differences and other variables in the usage of Drug Eluting Stents (DES) and Bare Metal Stents (BMS).

## Materials

Data from 100704 stent implantations performed during 82304 interventions were prospectively collected in the German ALKK-registry (Arbeitsgemeinschaft Leitende Kardiologische Krankenhausärzte) from 1st quarter 2005 to 4th quarter 2009. In the present analysis, data from 28 centres in Germany which participated continuously during the whole period were included. The project started in 1992 as a prospective registry for quality control in PTCA. The registry collects data about indication, technical aspects, medication and hospital outcome including in-hospital complications. Since 2002, the registry is based on an obligatory quality control program that has been introduced in Germany, which requires and checks consecutive enrolment and the completeness of a core dataset. The data were collected electronically and transferred in anonymised form to the *Institut für Herzinfarktforschung* for editing and statistical analysis. The study is purely observational and was approved by the ethics committee of the Landesaerztekammer Rheinland-Pfalz. None of the authors has competing interests concerning scope and results of the analysis.

All consecutive documented stent implantations for ST-elevation myocardial infarction (STEMI), Non-ST-elevation-Acute Coronary Syndrome (NSTE-ACS), or stable Coronary Artery Disease (CAD) were included in the present analysis.

## Methods

### Statistical analyses

Patients’ baseline and angiographic characteristics for both sexes are presented as percentages and absolute values with regard to categorical variables and compared by Pearson chi-squared test and odds ratios with 95%-confidence intervals. The distribution of continuous variables is characterised by median and quartiles and compared between genders by Wilcoxon rank-sum test. The stent diameter and the number of stents per procedure is summarized by mean and standard deviation. These descriptive statistics are based on the available cases. As patients admitted multiple times cannot be identified in the data base, we considered different interventions to be independent.

The proportion of DES compared to all implanted stents is shown for men and women in categories of relevant factors. The 95%-intervals of odds ratios adjusted standard errors were calculated using the Taylor linearization technique to allow for clustering. The use of DES in categories of age and indication for PCI is visualised in bar charts and tested for interaction by the Breslow-Day test.

In order to adjust the effect of gender on the choice of a drug eluting stent for other determinants, the variables whose distributions differed significantly between men and women on the one hand and DES and BMS on the other hand as well as the significant interaction of age and gender were included in a multivariable logistic model. As multiple stents implanted during the same session strongly tended to be of the same type, generalized estimating equations assuming an exchangeable working correlation structure were applied and robust standard errors calculated for the odds ratios. For explanatory variables with missing information of more than 1%, conditional means, calculated by a regression on age, gender and indication for PCI, were used.

All *p*-values are the results of two-tailed tests. *P*-values ≤ 0.05 were considered significant. The statistical calculations have been performed using the SAS system release 9.3 on a personal computer (SAS Institute, Cary, NC, USA).

## Results

### Patient characteristics

Women were significantly older than men (71.9 vs. 66.7 years) and were more likely to suffer from diabetes (26.5% vs 20.2%). In contrast, men more often had previous CABG or PCI (13.0% vs. 8.2% and 34.7% vs. 28.2%), while there was no difference regarding renal failure (Table 1).

**Table 1:** Patient and procedural chracteristics

Variable	Women	Men	*P*-value	OR (95%-CI)
Number of procedures	22946	59358		
Patient history:				
Age [years]	71.9 (64.8 – 78.3)	66.7 (57.4 – 73.5)	<0.001	
Diabetes mellitus	26.5% (5868/22154)	20.2% (11537/57209)	<0.001	1.43 (1.38–1.48)
Previous CABG	8.2% (1885/22880)	13.0% (7721/59200)	<0.001	0.60 (0.57–0.63)
Previous PCI	28.2% (6419/22747)	34.7% (20432/58861)	<0.001	0.74 (0.72–0.76)
Renal disease	13.6% (2977/21943)	13.3% (7510/56639)	0.255	1.03 (0.98–1.07)
Presentation:				
STEMI	18.2% (4182/22946)	19.3% (11478/59358)	<0.001	0.95 (0.91–0.99)
NSTE-ACS	29.3% (6721/22946)	27.6% (16370/59358)		1.07 (1.04–1.11)
Stable CAD	52.5% (12043/22946)	53.1% (31510/59358)		1^a^
Cardiogenic shock	1.7% (388/22945)	1.5% (894/59355)	0.055	1.12 (0.99–1.27)
Symptoms of HF	5.6% (1296/22945)	5.0% (2952/59355)	<0.001	1.14 (1.07–1.22)
Center volume stents/year	1150 ± 613	1156 ± 623	0.364	
Target lesions:				
RCA	35.0% (8039/22946)	34.5% (20508/59358)	0.190	1.02 (0.99–1.05)
LAD	45.9% (10526/22946)	41.0% (24358/59358)	<0.001	1.22 (1.18–1.26)
CX	23.6% (5412/22946)	26.9% (15972/59358)	<0.001	0.84 (0.81–0.87)
Left main stem	2.2% (504/22946)	2.6% (1538/59358)	0.001	0.84 (0.76–0.93)
Bypass graft	1.8% (416/22946)	3.5% (2107/593258)	<0.001	0.50 (0.45–0.56)
Implanted stents per PCI	1.40 ± 0.75	1.42 ± 0.76	<0.001	
In-stent restenosis	6.5% (1499/22914)	7.3% (4300/59293)	<0.001	0.90 (0.84–0.95)
Complex stenosis (≥ B2)	66.6% (15032/22579)	68.3% (39860/58402)	<0.001	0.93 (0.90–0.96)

(*CABG* Coronary artery bypass grafting, *PCI* percutaneous coronary intervention, *CAD* coronary artery disease, *RCA* right coronary artery, *LAD* left anterior descending artery, *CX* left circumflex artery, *PCI* percutaneous coronary intervention, *HF* heart failure)

^a^Reference category

The presentation with STEMI, NSTEMI or stable CAD as well as cardiogenic shock and with or without signs of heart failure, showed statistically significantly different but numerically similar values between genders. The same holds true for the lesion characteristics, where we found more left anterior descending (LAD) lesions and fewer left circumflex (CX) lesions, stent re-stenosis and complex lesions in women than in men. The centre experience in terms of stent implantations performed per year was comparable for men and women.

### Usage of DES from 2005 to 2009

**Fig. 1:**
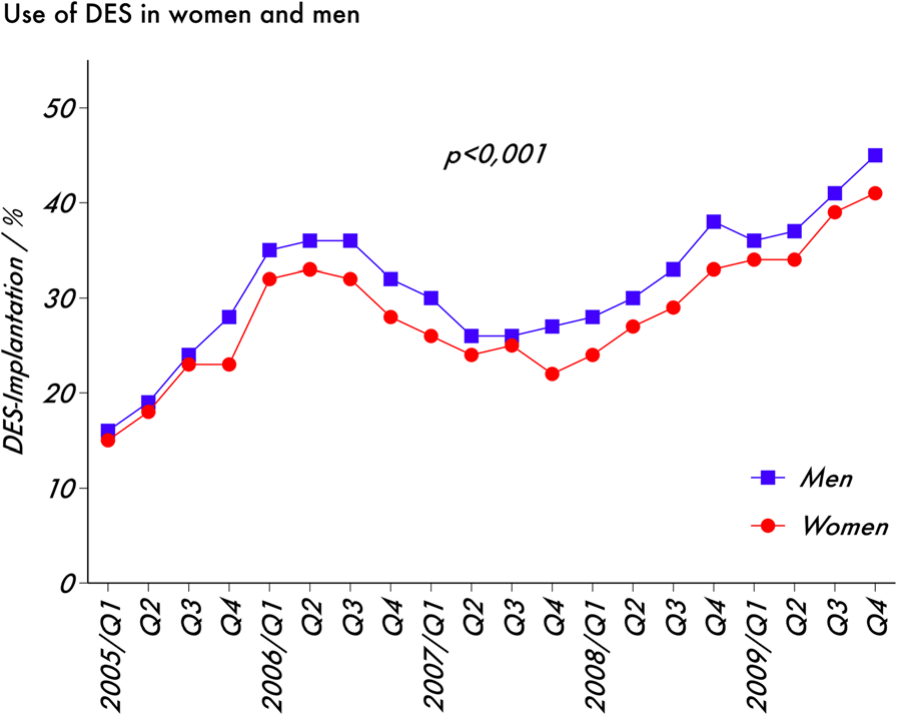
Percentage of DES use in women and men between 1^st^ quarter 2005 (Q1) until 4^th^ quarter 2009 (Q4)

Between 1^st^ quarter 2005 and 4^th^ quarter 2009, the use of DES increased from 16.0% to 43.9%. After a rapid increase from 2005 to early 2006, the implantation rate reached a plateau and decreased thereafter. Beginning with the 1^st^ quarter 2008, the rate of DES Implantation steadily increased until the end of the observation period. For all quarters of a year that have been analysed, women received lower rates of DES (*p* < 0.001; Fig. 1).

### Stent diameter

**Fig. 2:**
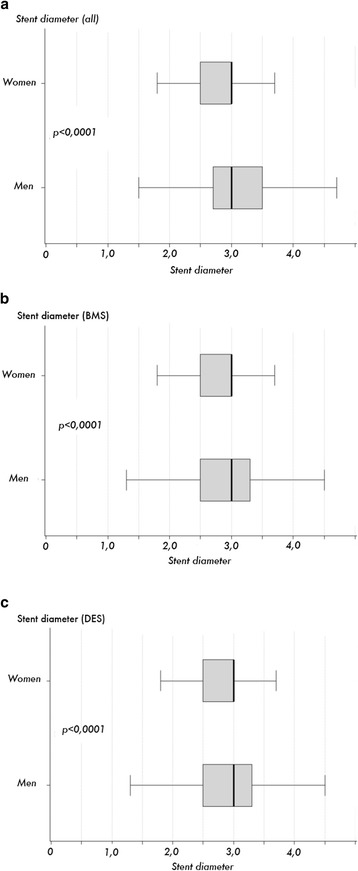
Box plots of stent diameter. Overall stent diameter (**a**) was larger in men than in women, as was stent diameter of BMS (**b**) and DES(**c**). Furthermore, overall DES diameter was smaller compared to BMS

Compared to men, women received stents with lower diameters, with a mean ± standard deviation of 2.94 ± 0.48 mm vs. 3.04 ± 0.53 mm (*p* < 0.0001) than in men (median 3.0 (quartiles 2.5-3.0) mm vs. 3.0 (2.7-3.5) mm, Fig. 2a). This difference between women and men was the same for BMS (2.96 ± 0.48 vs. 3.07 ± 0.53 mm respectively (*p* < 0.0001) (Fig. 2b), and DES, with a mean of 2.89 ± 0.48 vs. 2.97 ± 0.51 mm (*p* < 0.0001) (Fig. 2c).

### Relative use of DES in women and men according to indication

**Fig. 3:**
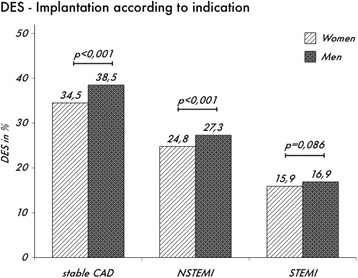
Relative amount of DES implantation rates in women and men for different age groups

All indications for stent implantation show a lower DES use in women compared to men. While for stable coronary artery disease and NSTEMI there was significant difference of 4.0 and 2.5% (*p* < 0.001) respectively, the use of DES in STEMI showed a trend (*p* < 0.086) with an absolute 1% lower use of DES in women (Fig. 3). However, no significant interaction was detected (*p =* 0.11).

### Age dependent rate of DES use in women and men

**Fig. 4:**
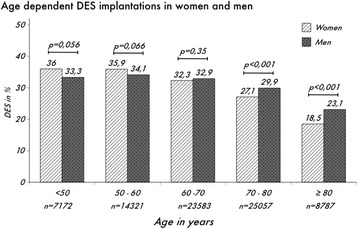
Percentage of DES use in stable CAD, NSTEMI- and STEMI-ACS

For the women under age 50 years and between 50 and 60 years, there was a trend (*p =* 0.056 and *p =* 0.066) to a higher use of DES in women compared to men, with a difference of 2.7 and 2.6%, respectively. In the age group between 60 and 70 years, there is a non significant difference of 0.7%, while women between 70 and 80 years and over 80 years receive fewer DES compared to men, 2.8% (*p* < 0.001) and 4.6% (*p* < 0.001) respectively. The age group over 70 years comprises over 42% of the stent implantations that were analysed (Fig. 4). The interaction was highly significant (*p* < 0.0001).

### Variables correlating with use of DES

In both genders, heart failure and renal disease are related to the predominant use of BMS instead of DES (Table 2), while diabetes and previous PCI or CABG were associated with a higher use of DES. Concerning the treated lesions, in-stent restenosis and complex anatomy as well as left main stem or LAD locations correlated with greater DES use.

**Table 2:** Use of DES in women and men

Variable	Women		Men	
	DES [%]	OR (95%-CI)	DES [%]	OR (95%-CI)
Total stents	28.2% (7857/27891)		31.3% (22779/72813)	
Patient history:				
Previous PCI yes/no	43.9/22.3	2.74 (2.56–2.93)	44.7/24.5	2.49 (2.39–2.59)
Previous CABG yes/no	36.3/27.5	1.50 (1.35–1.67)	38.2/30.3	1.42 (1.35–1.50)
Diabetes mellitus yes/no	32.6/26.6	1.34 (1.25–1.43)	36.3/30.3	1.31 (1.25–1.37)
Renal disease yes/no	23.7/29.5	0.74 (0.67–0.82)	28.6/32.5	0.83 (0.78–0.88)
Cardiogenic shock yes/no	9.6/28.5	0.27 (0.18-0.38)	13.7/31.6	0.34 (0.28-0.42)
Symptoms of HF yes/no	16.8/28.9	0.50 (0.42-0.59)	21.3/31.8	0.58 (0.53-0.64)
Lesion characteristics:				
RCA	24.9	1*	26.9	1*
LAD	31.8	1.41 (1.32-1.51)	35.8	1.51 (1.45-1.58)
CX	24.2	0.96 (0.89-1.04)	28.9	1.10 (1.05-1.15)
LMCA	45.9	2.56 (2.13-3.08)	50.1	2.73 (2.45-3.03)
Bypass graft	31.2	1.37 (1.12-1.67)	30.0	1.16 (1.06-1.27)
In-stent restenosis yes/no	76.6/25.4	9.62 (8.45-10.95)	75.0/28.5	7.55 (7.00-8.14)
Complex stenosis (≥ B2)	30.9/23.8	1.43 (1.34-1.52)	33.6/27.4	1.34 (1.29-1.39)

All comparisions vs. RCA (as reference), except for CX in woman were sigificant (*p*<0,05). (*CABG* coronary artery bypass grafting, *PCI* percutaneous coronary intervention, *CAD* coronary artery disease, *RCA* right coronary artery, *LAD* left anterior descending artery, *CX* left circumflex artery, *PCI* percutaneous coronary intervention, *HF* heart failure, *LMCA* left main coronary artery)

*Reference category

In the multivariable model, diabetes was a strong predictor of DES use (OR 1.39, *p* < 0.001), while STEMI (OR 0.34, *p* < 0.001), cardiogenic shock (OR 0.56, *p* < 0.001), NSTEMI-ACS (OR 0.61, *p* < 0.001), stent diameter (OR 0.67 for every mm increase in stent diameter, *p* < 0.001) and age (OR 0.80 for every 10 years increase in age) and female gender (above the age of 75) were all associated with a lower usage of DES. As a significant interaction between age and gender has been detected (*p* < 0.0001), the effect estimates of age are reported separately for men and women, and those of female gender for distinct age values (Table 3). When we fitted the regression model separately in the subgroups of stable CAD, NSTE-ACS and STEMI, the adjusted effect of female gender on the use of DES was strongest in patients with stable CAD (OR 0.88 (0.82-0.94) at age 80 years), weaker in NSTE-ACS (OR 0.90 (0.80-0.99)) and insignificant in STEMI (OR 1.00 (0.85-1.18)).

**Table 3:** ALKK PCI-registry 2005-2009: Adjusted effects for the usage of DES in all stent implantations (*n* = 29374/97491)

Explanatory variable	*P* value	Adjusted odds ratio	95%-CI
Age [10-year increase] in men	<0.001	0.81	0.79-0.82
Age [10-year increase] in women	<0.001	0.74	0.72-0.76
Female sex at age 75 years	0.002	0.93	0.89-0.97
Female sex at age 80 years	<0.001	0.89	0.84-0.94
Diabetes	<0.001	1.29	1.24-1.35
STEMI vs. elective	<0.001	0.37	0.36-0.39
NSTEMI vs. elective	<0.001	0.64	0.61-0.66
Cardiogenic Shock	<0.001	0.46	0.38-0.55
Moderate symptoms of HF	<0.001	0.72	0.66-0.80
Previous PCI	<0.001	1.80	1.74-1.87
Previous CABG	<0.001	1.27	1.21-1.34
LAD	<0.001	1.63	1.57-1.68
Left main stem	<0.001	2.66	2.41-2.93
In-stent restenosis	<0.001	5.63	5.28-6.01
Complex stenosis (≥ B2)	<0.001	1.54	1.49-1.60
Stent diameter [for every mm]	<0.001	0.81	0.79-0.84

(*CABG* Coronary artery bypass grafting, *PCI* percutaneous coronary intervention, *CAD* coronary artery disease, *RCA* right coronary artery, *LAD* left anterior descending artery, *CX* left circumflex artery, *PCI* percutaneous coronary intervention, *HF* heart failure, *STEMI* ST-elevation myocardial infarction, *NSTEMI* non ST-elevation myocardial Infarction)

### Adjuvant medical therapy, major adverse cardiac and cerebrovascular event (MACCE) and access site complications

Medical therapy including platelet inhibition and anticoagulation in patients aged 70 years and older did not show a clinically relevant difference between women and men. However, in-hospital mortality and MACCE were significantly higher in women. Most notably, the difference was driven by a significant difference in stable CAD (Table 4). Non-MACCE related access site complications, predominantly bleeding, were more common in women than in men; the difference was significant for all indications (Table 4).

**Table 4:** Adjuvant medical therapy, MACCE and access site complications in women and men ≥ 70 years

		Women	Men	*P*-value
Medical therapy during PCI				
Heparin (%)	Total	85.5	84.8	0.160
	STEMI	90.7	91.0	0.710
	NSTEMI	85.2	84.0	0.184
	stable CAD	83.8	83.4	0.606
LMW-Heparin (%)	Total	3.4	3.7	0.211
	STEMI	4.9	4.4	0.461
	NSTEMI	5.7	6.7	0.080
	stable CAD	1.5	1.9	0.065
Bivalirudin (%)	Total	0.5	0.6	0.281
	STEMI	0.4	0.4	0.730
	NSTEMI	0.9	0.8	0.893
	stable CAD	0.4	0.6	0.113
ASA i.v. (%)	**Total**	**43.9**	**41.6**	**<0.001**
	STEMI	66.0	67.1	0.434
	**NSTEMI**	**42.1**	**39.3**	**0.025**
	stable CAD	36.9	35.4	0.093
ASA oral (%)	**Total**	**62.2**	**63.7**	**0.019**
	STEMI	46.1	47.7	0.313
	NSTEMI	65.0	65.2	0.834
	stable CAD	66.3	67.5	0.169
Clopidogrel (%)	Total	88.5	88.4	0.880
	STEMI	91.7	92.1	0.678
	NSTEMI	90.8	90.6	0.832
	stable CAD	86.0	86.3	0.647
GPIIb/IIIa-Inhibitor (%)	Total	21.8	21.5	0.645
	STEMI	57.4	59.7	0.085
	NSTEMI	26.1	27.3	0.171
	**stable CAD**	**7.1**	**8.3**	**0.002**
Procedure related mortality and MACCE				
Mortality (intrahospital) (%)	**Total**	**2.8**	**2.2**	**0.006**
	STEMI	9.4	8.4	0.251
	NSTEMI	2.9	2.7	0.686
	**stable CAD**	**0.6**	**0.3**	**0.013**
MACCE (Death, MI, Stroke/TIA) (%)	**Total**	**3.3**	**2.8**	**0.023**
	STEMI	10.2	9.3	0.399
	NSTEMI	3.5	3.2	0.452
	**stable CAD**	**1.2**	**0.8**	**0.031**
Non-MACCE access site related complications (i.e. bleeding) (%)	**Total**	**3.6**	**1.8**	**<0.001**
	**STEMI**	**4.2**	**1.3**	**<0.001**
	**NSTEMI**	**4.0**	**2.1**	**<0.001**
	**stable CAD**	**3.3**	**1.8**	**<0.001**

(*ASA* acetylsalicylic acid, *LMW* low molecular weight, *CABG* Coronary artery bypass grafting, *PCI* percutaneous coronary intervention, *CAD* coronary artery disease, *STEMI* ST-elevation myocardial infarction, *NSTEMI* non ST-elevation myocardial Infarction, *MACCE* major adverse cardiac and cerebrovascular event)

Significant values are presented in bold

## Discussion

The main finding of our analysis is a lower rate of DES in elderly women, which is not in accordance with contemporary guidelines on revascularisation [14].

### Gender, vessel size and DES use

Correlated with a smaller body surface area [15], women have smaller diameter coronary arteries than men, which explains the inferior results in revascularisation procedures, either PCI [5] or CABG [6]. In the ALKK (Arbeitsgemeinschaft Leitende Kardiologische Krankenhausärzte)-PCI registry we found that stents used in women were smaller than those used in men, either for BMS and DES (Fig. 2a, b, c). These data indirectly confirm, that women have smaller epicardial vessels. For both genders, DES were more frequently used in smaller vessels, which reflects the fact that our data were derived from 2005 to 2009, before large data on the use of DES in larger vessels were available [16, 17]. The tendency to use DES in smaller vessels suggests women may receive a predominance of DES compared to men. However, univariate analysis showed that women received a lower percentage of DES compared to men between 2005 and 2009 (Fig. 1). Further analysis in a multivariable logistic model revealed that the lower likelihood for women to receive a DES is observed only in women above the age of 75 year.

The finding of less frequent DES use in women were also evident irrespective of different indications for PCI, like stable angina, NSTEMI and STEMI. However, while the difference in the two former were statistically significant, there was only a trend towards a lower usage of DES in the latter (Fig. 3). The higher frequency of DES implantation in stable disease compared to ACS reflects data progression suggesting superiority of DES even in STEMI-ACS [18]. This benefit of DES use in ACS is confined to reduced repeat target revascularisation, rather than lower mortality [19].

### Explanations for the lower use of DES in elderly women

The underuse of DES is an unexpected finding with different possible explanations:

First, the lower rates of DES in older women could be a chance finding. However, the large number of stent implantations and the high significance (*p* < 0,001) render this explanation unlikely. Furthermore, the adjusted effects show a higher usage of DES in diabetes, whereas ACS and cardiogenic shock were correlated with a lesser use of DES (Table 1). These results are all quite expected and confirm the plausibility of the database.

Another explanation could be an unknown confounder accounting for the findings. Concerns exist regarding DES (and hence dual antiplatelet therapy) use where there is the need for oral anticoagulation, (such as after implantation of a mechanical heart valve or as a result of repeated thrombo-embolic disease). A commonly encountered scenario is dual antiplatelet therapy (DAPT) in addition to oral anticoagulation for patients with atrial fibrillation. The burden of atrial fibrillation is unlikely to explain the disparity in DES use in women over 75 compared to men over 75 however because although information about long term anticoagulation or about atrial fibrillation are not available in our database, the Framingham heart study [20] suggests atrial fibrillation is 1.5 times more common in men over 75 compared to women over 75 so this in itself is unlikely to explain lower DES use in women of this age. Operators may attribute a higher risk of bleeding to elderly women, which is based on objective data on higher peri-procedural bleeding complications [21] and a higher prevalence of anemia [22], that are also predictors of long term mortality [23], as well as a subjective perception of frailty in elderly women.

Therefore, even if peri-procedural bleeding complications do not differ depending on the stent used, the awareness of a higher liability for bleeding could have encouraged interventionalists to rather use BMS instead of DES, whenever there is a suspected risk of bleeding complications, which is typically encountered in elderly women. Interestingly, the tendency to use BMS instead of DES is confined to the type of stent, since anti-platelet therapy and anticoagulation do not differ (Table 4). Especially the use of GPIIb/IIIa inhibitors, which are known for a higher rate of periprocedural bleeding complications [24] (Table 4), was similar in both genders.

Actually, our study (Table 4) confirms a substantially higher periprocedural risk of access site complication, bleeding, MACCE and death in women [15, 25, 26]. Given the higher probability of target lesion revascularisations for in stent restenoses with

BMS [27], elderly women are likely exposed to a higher overall risk due to repeat revascularisation procedures.

Paradoxically, the intention to prevent bleeding complications in women by the use of BMS instead of DES, could actually increase morbidity and mortality.

There could be doubts regarding the efficacy of DES in women, (as women are thought to have less complex coronary lesions [28] which could be treated equally with BMS or DES), particularly as DES are more expensive than BMS. Indeed, Our data shows, a lower percentage of complex lesions and in-stent restenosis in women comared to men. While there is a lack of improvement for mortality and MI, adequately powered RCTs impressively demonstrated a reduction of target vessel revascularisation with DES compared to BMS, which were similar for both sexes [28], even in the elderly [29]. These results were confirmed by registry data on the use of DES, that show that these findings proved to be valid in daily practice [30].

The data from the PCI registry show, that women receive lower percentage of DES compared to men; this difference is significant only for the age groups over 70 years (Fig. 4). However, the age group between 70 and 80 years is the largest in the registry and overall, people older than 70 years account for more than 40% of the sample. Given the high number of PCIs analysed, these data are considered to be clinically relevant, showing that women do not receive best available treatment for coronary artery disease.

### Duration of dual anti-platelet therapy

The duration of DAPT was not included in our database, however, according to guidelines, patients with STEMI and NSTEMI received DAPT for 12 month, independent of stent type, while patients with stable CAD received DAPT for one or six month for BMS and DES, respectively. Actually, we found the biggest difference in stent usage in the group with stable angina. Given there are more bleedings, this could explain the tendency to prefer BMS in elderly woman in the era of 1st generation DES.

However, latest data for 2nd generation DES show good results for short DAPT, favouring a DAPT for only three month [31] in patients with concerns of bleeding. This shortening of DAPT should further reduce the difference in DES use between men and women.

### Limitations

Our large sample, which is representative for current PCI-procedures in Germany provides data on the use of DES and BMS in women and men in different revascularisation settings. However, our study has some limitations.

First, whilst we adjusted for baseline differences, we cannot fully eliminate them. As a result, it is possible that unmeasured confounders (especially atrial fibrillation and oral anticoagulation therapy requirement) exist and may have contributed to the differences observed. Second, the analysis that was performed is retrospective and is, therefore, dependent on the data already collected.

## Conclusion

The ALKK-PCI database shows a distinctly lower use of DES in elderly women. This difference is not supported by guidelines or recent published trials. As undertreatment cannot be studied by randomised controlled trials, other available databases on PCI should be analysed for gender differences in DES implantation.

More effort should be made to uniformly implement of current revascularisation guidelines across groups, thereby eliminating gender differences in care.

## Abbreviations

ACS: Acute coronary syndrome
ALKK: Arbeitsgemeinschaft Leitende Kardiologische Krankenhausärzte
BMS: Bare metall stent
CABG: Coronary artery bypass grafting
CAD: Coronary artery disease
CX: Left circumflex artery
DAPT: Dual antiplatelet therapy
DES: Drug eluting stent
HF: Heart failure
ISR: In-stent restenosis
LAD: Left anterior descending artery
MACCE: Major adverse cardiac and cerebrovascular event
NSTEMI: Non ST-elevation myocardial Infarction
OR: Odds ratio
PCI: Percutaneous coronary intervention
RCA: Right coronary artery
RCT: Randomized controlled trial
STEMI: ST-elevation myocardial infarction
